# Identification of source and sink populations for the emergence and global spread of the East-Asia clone of community-associated MRSA

**DOI:** 10.1186/s13059-016-1022-0

**Published:** 2016-07-26

**Authors:** Melissa J. Ward, Mariya Goncheva, Emily Richardson, Paul R. McAdam, Emma Raftis, Angela Kearns, Robert S. Daum, Michael Z. David, Tsai Ling Lauderdale, Giles F. Edwards, Graeme R. Nimmo, Geoffrey W. Coombs, Xander Huijsdens, Mark E. J. Woolhouse, J. Ross Fitzgerald

**Affiliations:** 1 Centre for Immunity, Infection and Evolution, University of Edinburgh, Edinburgh, UK; 2 The Roslin Institute, University of Edinburgh, Edinburgh, UK; 3 Public Health England, Colindale, UK; 4 University of Chicago, Chicago, IL USA; 5 National Health Research Institutes, Zhunan, Taiwan; 6 Scottish MRSA Reference Laboratory, NHS Greater Glasgow and Clyde, Glasgow, UK; 7 Griffith University School of Medicine, Gold Coast, QLD Australia; 8 Australian Collaborating Centre for Enterococcus and Staphylococcus Species (ACCESS) Typing and Research, Murdoch University, Perth, Australia; 9 National Institute for Public Health and the Environment, Bilthoven, The Netherlands

## Abstract

**Background:**

Our understanding of the factors influencing the emergence, dissemination and global distribution of epidemic clones of bacteria is limited. ST59 is a major epidemic clone of community-associated MRSA in East Asia, responsible for extensive morbidity and mortality, but has a much lower prevalence in other parts of the world. The geographic origin of ST59 and its international routes of dissemination are unclear and disputed in the literature.

**Results:**

To investigate the origin and spread of the ST59 clone, we obtained whole genome sequences of isolates from four continents, sampled over more than a decade, and carried out a time-scaled phylogeographic analysis. We discover that two distinct ST59 clades emerged concurrently, in East Asia and the USA, but underwent clonal expansion at different times. The East Asia clade was strongly enriched for gene determinants associated with antibiotic resistance, consistent with regional differences in antibiotic usage. Both clones spread independently to Australia and Europe, and we found evidence of the persistence of multi-drug resistance following export from East Asia. Direct transfer of strains between Taiwan and the USA was not observed in either direction, consistent with geographic niche exclusion.

**Conclusions:**

Our results resolve a longstanding controversy regarding the origin of the ST59 clone, revealing the major global source and sink populations and routes for the spread of multi-drug resistant clones. Additionally, our findings indicate that diversification of the accessory genome of epidemic clones partly reflects region-specific patterns of antibiotic usage, which may influence bacterial fitness after transmission to different geographic locations.

**Electronic supplementary material:**

The online version of this article (doi:10.1186/s13059-016-1022-0) contains supplementary material, which is available to authorized users.

## Background

Since the 1990s, methicillin-resistant *Staphylococcus aureus* (MRSA) strains have been identified outside the hospital setting, often in otherwise healthy individuals with no obvious recent healthcare-associated risk factors (reviewed in [1]). These strains, termed ‘community-associated’ MRSA (CA-MRSA), are suggested to represent a pool of strains which are distinct from healthcare-associated MRSA (HA-MRSA) and have been found to possess novel SCC*mec* types (IV and V rather than I–III) [2, 3] and to encode toxins such as Panton-Valentine leukocidin (PVL) [4, 5].

Whilst many CA-MRSA clones have a global distribution, the considerable variation in the prevalence and dominance of clones between different geographical niches remains unexplained. For example, in the USA, the ST8 USA300 clone is the dominant epidemic clone, responsible for very high levels of human disease and mortality over more than a decade [6, 7]. In contrast, *S. aureus* clonal complex 59 (CC59) is a major CA-MRSA clone in East Asia and is the dominant clone in Taiwan [8–10], where it is a major cause of skin and soft tissue infections [11] and has also been identified as a widespread commensal coloniser (discussed in [12]). CC59 colonisation or infection has been reported in many countries globally, including the USA [6, 13], where the USA1000 ST59 strain is found, but the prevalence is much lower compared with East Asia.

Previous studies have devised groupings for CC59 strains according to variation in SCC*mec* elements and carriage of PVL, with one such group being the virulent ‘Taiwan clone’ which included ST59, ST338 and ST952 isolates [14]. In addition to being PVL-positive, the Taiwan clone has been shown to possess a novel type V SCC*mec* element, known as V[5C2&5] or V_T_ [15]. A recent comparative genomics study using microarray data has indicated that Taiwanese ST59 strains may be distinguished between the Taiwan clone and a distinct, commensal ‘Asia-Pacific’ clone (SCC*mec* type IV, PVL-negative) on the basis of carriage of specific mobile genetic elements [12]. The prevalence of multi-drug resistance amongst both skin and soft tissue infection and commensal ST59 isolates in Taiwan has been reported to be unusually high compared with other CA-MRSA strains [16].

Phylogenetic studies across the whole diversity of *S. aureus* have speculated upon the ancestral origins of CC59, suggesting that it emerged through a host jump from livestock into humans at least 500 years ago [17, 18]. The global spread of CC59 strains has also been the subject of controversy in the literature, with suggestions that ST59 originated in the USA and spread eastwards towards Asia and/or that there has been spread of ST59 out of Taiwan [19, 20]. Proposals regarding the geographical origins of ST59 strains are based upon case reports and multi-locus sequence typing of CA-MRSA isolates and are thus highly influenced by the order in which isolates are reported. For example, the USA was the only country from which ST59 was found in a 2003 study of CA-MRSA strains; along with the apparent finding of continent-specific clones, this led researchers to conclude that CA-MRSA ST59 originated in the USA [21]. Subsequent reports of CA-MRSA ST59 isolated in Taiwan between 1997 and 2002 [22], followed by its discovery in Singapore, the Netherlands and France, indicated that CA-MRSA has spread at the inter-continental level and led some researchers to conclude that exchange of USA1000 ST59 had occurred from the USA towards Asia [23], although this remains a point of contention [19].

To date, little is known about the relative timings of the expansions of the USA1000 and Taiwan ST59 clones. Using whole-genome phylogeographic analyses, we formally test hypotheses about the spread of ST59 between different countries or continents and investigate the evolutionary relationships between them. We describe the spatiotemporal spread of CC59 using a dataset of 120 global CC59 genomes, investigate the distribution of mobile genetic elements in CC59 strains from around the world and consider the potential influence of inter-clone competition on the global distribution of CA-MRSA strains.

## Results

### Distinct clades of CC59 originated in USA and East Asia

**Fig. 1:**
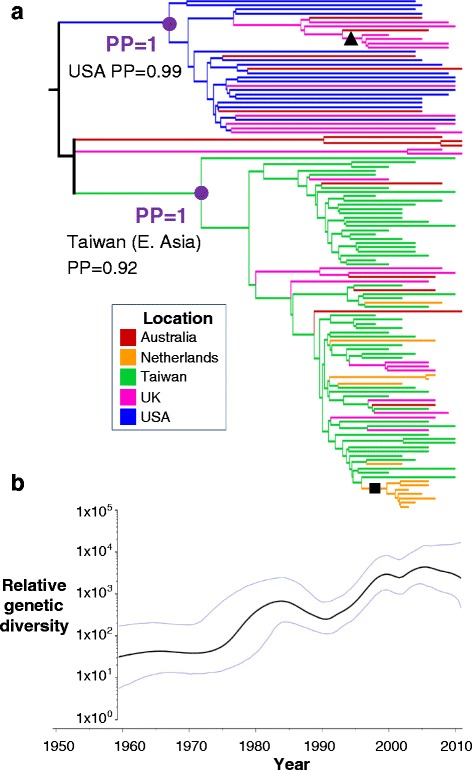
Maximum clade credibility (MCC) tree showing **a** the phylogenetic relationships between global CC59 sequences and **b** the relative genetic diversity of global CC59 over time. The MCC tree is summarised from 9000 BEAST posterior phylogeny samples. *Branches* are coloured according to the inferred ancestral location under a discrete phylogeography analysis. Due to low power at the root of the tree, we were unable to infer the ancestral location with a posterior probability greater than 0.5 (*branches coloured black*). *Purple dots* denote the ancestral nodes of two major clades, one which was inferred to have originated in the USA and the other in East Asia. A *black triangle* denotes a clade of sequences from the UK found within the USA-associated clade and a *black square* denotes a clade of sequences from the Netherlands found within the East Asia clade. Median and 95 % highest posterior density (HPD) intervals for the relative genetic diversity of CC59 over time are plotted against time, with the lower limit of the timescale corresponding to the lower 95 % HPD value of the root height of the tree. The phylogeny and the plot of the relative genetic diversity are shown on the same timescale. *PP* posterior probability

We carried out whole-genome sequencing of 120 human CC59 isolates from Australia, the Netherlands, Taiwan, the UK and the USA, sampled between 1998 and 2011, including reference isolates (Additional file 1: Figure S1, Figure S2 and Table S1). Two major clades, each with a posterior probability of 1, were identified in a time-scaled BEAST phylogeny constructed from the core genome (Fig. 1a). One clade consisted of sequences from the USA, Australia and the UK, with the other comprising sequences from Taiwan, Australia, the UK and the Netherlands. Notably, while isolates from Europe and Australia were found in both clades, we observed a clear phylogenetic separation between CC59 strains from Taiwan and the USA. The same split into two major clades was observed in a maximum likelihood phylogenetic analysis of core genome sequences (Additional file 1: Figure S3).

There was considerable difficulty in placing a group of five sequences (three from Australia and two from the UK) in the BEAST analysis, as indicated by low posterior support for their position in the tree (Additional file 1: Figure S4). The presence of these five sequences in the analysis did not affect our overall phylogenetic conclusions (emergence dates and composition of the two major clades remained the same when the analysis was run without them).

Phylogeographic mapping of the country of sampling onto the BEAST phylogeny samples indicated the clade containing Taiwanese isolates had its ancestral location in Taiwan (posterior probability (PP) = 0.92) and that the other major clade had its ancestral location in the USA (PP = 0.99). We henceforth refer to these clades as the ‘East Asia clade’ and the ‘USA clade’, respectively. The East Asia and USA clades emerged around the same time: 1966 for the USA clade (95 % highest posterior density (HPD) interval = [1959, 1973]); 1971 for the East Asia clade (95 % HPD interval = [1965, 1977]). However, plots of the relative genetic diversity of CC59 over time are consistent with the hypothesis that the expansion of the USA clade preceded the expansion of the East Asia clade by approximately 10 years (Fig. 1a, b).

### CC59 has spread on an intercontinental scale but with geographical restriction between Taiwan and the USA

Isolates from Australia and Europe were interspersed across both the USA and East Asia clades, suggestive of frequent inter-continental spread of CC59. Within the East Asia clade we observed a sub-clade (PP = 1; denoted by black square in Fig. 1a) of seven sequences from the Netherlands, sampled between 2003 and 2007 and sharing a common ancestor around 2000. Similarly, within the USA clade, a sub-clade of five sequences sampled in the UK between 2000 and 2009 which shared a common ancestor in the mid-1990s was observed (PP = 1; denoted by black triangle in Fig. 1a), suggesting that ST59 is able to be maintained and cause outbreaks following dissemination from East Asia or the USA.

**Fig. 2:**
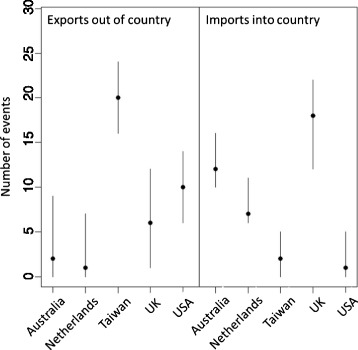
Inferred number of CC59 imports and exports. The median number of transitions out of and into each country, as inferred using a Markov jumps analysis, was calculated across the BEAST phylogeny samples. The 95 % HPD intervals are shown as *lines*, with medians indicated by *dots*. Australia, the Netherlands and the UK were inferred to be sink populations for ST59, with Taiwan/East Asia and the USA as source populations

**Fig. 3:**
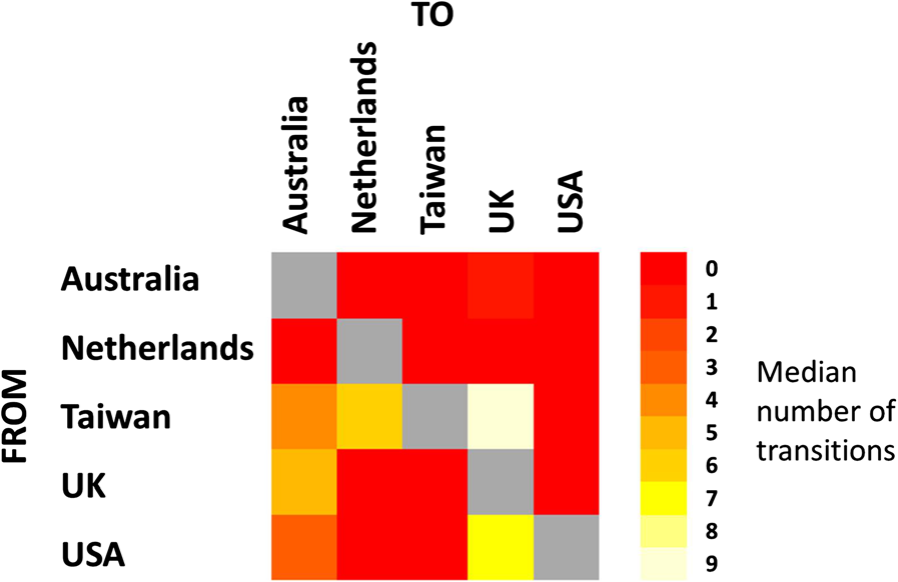
Inferred number of transitions between locations. The median number of transitions from one location to another under the Markov jumps analysis was calculated across the BEAST phylogeny samples. *Lighter blocks* in the heat map indicate a higher number of transitions. The highest numbers of transitions were from Taiwan to Australia and the UK and from the USA to the UK

We used discrete trait mapping ‘Markov jumps’ methods in BEAST to summarise the number of transitions into and out of individual countries and identify major exporters and importers of CC59 (Fig. 2). We observed significantly more transitions into the UK, the Netherlands and Australia than into the USA and Taiwan. In contrast, the highest number of exports was out of Taiwan. On the basis of these analyses, Australia, the UK and the Netherlands were identified as sink populations for ST59, whilst Taiwan and the USA were identified as source populations. The median number of transitions from one country to another was calculated across the BEAST phylogeny samples and plotted as a heat map (Fig. 3). The highest numbers of transitions, and the only directed links for which the lower 95 % HPD limit was greater than 0, were from Taiwan into the UK (median number of transitions = 9, 95 % HPD interval = [6, 13]), from Taiwan into the Netherlands (median number of transitions = 6, 95 % HPD interval = [4, 9]), and from the USA into the UK (median number of transitions = 6, 95 % HPD interval = [2, 11]).

Bayes factor support for links between countries in a parsimonious description of the dissemination of CC59 was assessed through a Bayesian stochastic search variable selection (BSSVS) analysis. Strongly supported links (Bayes factor greater than 20) were found between the Taiwan and the Netherlands, between Taiwan and the UK and between Australia and the UK. The most strongly supported link involving the USA was between the USA and Australia; this was the fourth most strongly supported link in all independent BEAST runs, with a Bayes factor of approximately 3.

We assessed the robustness of results from the phylogeographic analysis to sampling effects by re-running the BEAST analyses on multiple down-sampled CC59 sequence datasets. In half of the runs we sampled from all countries across the entire period for which data were available (1998–2011), whilst in the other half we only sampled sequences from 2002 or later, as there were few sequences from countries other than Taiwan prior to 2002. The results from the subsampling analysis were highly consistent with our findings from the full dataset (Additional file 1: Figures S5–S9). Overall, these data implicate East Asia and the USA as the main global reservoirs for CC59, and we can identify major routes of intercontinental dissemination from those countries to Australia and Europe. Our data do not demonstrate transmission between Taiwan and the USA, in either direction, contrary to published assertions regarding the spread of CC59 between those countries [23]. Analysis of five independent phylogeography runs was not able to reliably estimate the geographical location at the root of the tree, with different locations being identified as the most probable ancestral location in different runs and always with a low PP (lower than 0.5).

### CC59 isolates from USA and Taiwan have accessory genomes with distinct antibiotic resistance gene profiles

In order to test the hypothesis that there were differences in gene content between the East Asia and USA clades which reflected their distinct geographic origins, we performed pangenomic analysis of all 120 CC59 isolates, identifying a total of 1420 accessory genes (i.e. genes not found in all isolates). We analysed the distribution of the accessory genes across the core genome phylogeny of the 120 CC59 sequences and used agglomerative hierarchical clustering to group the strains according to the presence or absence of accessory genes. We found that the most basal split into two clusters corresponded exactly with membership of the East Asia or USA clades in the core genome phylogeny (Additional file 1: Figure S10). Under Fisher’s exact test with a multiple testing correction, 153 genes were identified as significantly differing in presence or absence between the USA and East Asia clades. Only minor variation was observed in the number of core and accessory genes identified when different sequence similarity cutoffs (70, 80 or 90 %) were used in the pangenome analysis.

For genes identified as associated with presence in either the East Asia or USA clade and absence in the other, we noted whether there was evidence in the literature of involvement in antibiotic resistance or bacterial virulence or pathogenicity. We also looked for genes associated with competition which differed significantly in their presence between the USA and East Asia clades to investigate potential differences between the clades which might account for the observed lack of transfer of ST59 between the USA and Taiwan. From the Prokka annotation we identified a bacteriocin biosynthesis operon which was present in 75 % of isolates in the USA clade and 0.08 % of isolates in the East Asia clade. The encoded bacteriocin sequence (97 amino acids in length) was identical at the amino acid level for all CC59 isolates in our study in which it was found. BLAST searches found matches to the sequence with a very high level of coverage and similarity (both greater than 90 %) amongst *S. aureus* genomes from GenBank, as well as to an apparent variant (approximately 90 % coverage and 50 % sequence identity), both of which were found amongst USA300 strains. The existence of other genes in the same genomic region, with identical patterns of presence or absence across the CC59 isolates, suggests multiple genes have been imported or lost simultaneously with the bacteriocin. This is consistent with the bacteriocin being encoded on a plasmid, as was observed for publicly available genomes, including some belonging to USA300 (e.g. CP002146.1).

**Fig. 4:**
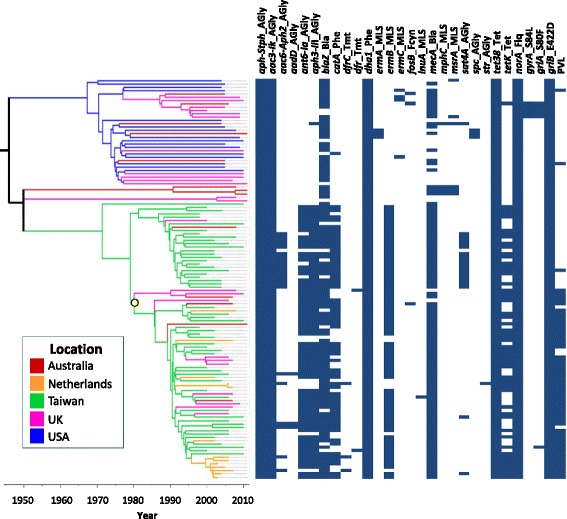
Presence or absence of antibiotic resistance genes amongst global CC59 isolates. SRST2 was used to map the raw sequencing reads for CC59 isolates against the ARG-ANNOT database of genes known to be associated with antibiotic resistance. The distribution of genes is plotted against the core genome phylogeny, with presence of a gene indicated by a *blue rectangle. Column names* are in the form “X_Y”, where X is the abbreviated name of the gene, and Y is the abbreviated name of the class(es) of antibiotic to which the gene is associated with resistance. The presence of amino acid variants associated with ciprofloxacin resistance is plotted, with resistant genotypes indicated in *blue* (e.g. A_BCD, where A is the gene name, C is the position of the variant amino acid, B is the residue of the susceptible wild type and D is the residue of the resistant type). Presence of Panton-Valentine leukocidin (PVL) is also indicated by *blue rectangles* and a subclade of the East Asia clade associated with PVL carriage is indicated by a *yellow dot*

In addition to the assembly-based pangenome analysis described above, we mapped the sequencing short reads to gene sequences in reference databases for *S. aureus* antibiotic resistance. This allowed us to assess the presence and absence of known resistance genes for each CC59 isolate. Consistent with the pangenome analysis, the mapping analysis revealed six antibiotic resistance genes (*catA*, *ermB*, *mecA*, *tetK*, *ant(6)-Ia*, *aph(3)-III*) which were significantly associated with presence in the CC59 isolates from Taiwan and absence in CC59 isolates from the USA, whilst no resistance genes were associated with presence in the USA isolates and absence in the Taiwan isolates (Fig. 4 and Table 1). Our data suggest that, in contrast to most isolates in the USA clade, the Taiwanese isolates have acquired genes conferring resistance to beta-lactams, chloramphenicol, tetracyclines and macrolides (or that the genes were lost from the USA clade but not from the East Asia clade).

**Table 1:** Antibiotic resistance genes associated with presence in the East Asia ST59 clade and absence in the USA ST59 clade

Gene associated with presence in East Asia clade and absence in USA clade	Antibiotic(s) to which resistance conferred
*catA*	Chloramphenicol
*ermB*	Macrolides, lincosamides, streptogramins B
*mecA*	Beta-lactams
*tetK*	Tetracyclines
*ant(6)-Ia*	Aminoglycosides
*aph(3)-III*	Aminoglycosides

Presence of genes was determined by mapping short read sequence data to a database of reference genes using the method described in [57]. The distribution of antibiotic resistance genes across the CC59 isolates is depicted in Fig. 4

Within the USA and East Asia clades, some variation could be observed between the antibiotic resistance profiles of isolates from different countries (Fig. 4). For example, only 31 % of USA isolates had the *mecA* gene and these strains were distributed across the USA clade, whereas a subclade of eight isolates from the UK and Australia within the USA clade all carried *mecA*, although such observations could be affected by sampling. Evidence was observed for the local acquisition of resistance determinants within countries: the only isolates in our study to possess amino acid variants in the *gyrA* and *grlA* genes associated with ciprofloxacin resistance were a subclade of five isolates from Australia and the UK that fell within the USA clade. Analysis of the 95 % HPD intervals for the dates of the branch immediately preceding this clade yielded an estimate of 1983–1995 for the emergence of fluoroquinolone resistance in UK strains of ST59, concordant with phylogenetic estimates for healthcare-associated *S. aureus* in the UK [24], although with a wider HPD interval due to lower power (fewer resistant strains, from across a smaller timespan) in our dataset. Drug-resistant strains may also spread from one country to another. For example, whilst the *ermB* gene was absent in all isolates within the USA clade, including those from Europe and the USA, within the East Asia clade it was present in 93 % of isolates from Taiwan and 78 % of European and Australian strains, consistent with the spread of the resistant strains out of East Asia. Similarly, *catA* was absent in all but one isolate in the USA clade but present in 86 % of isolates from Taiwan and 74 % of isolates from Europe and Australia within the East Asia clade. There is evidence that resistance determinants may have been maintained and spread in local communities after export from Taiwan. For example, in a sub-clade of seven isolates from the Netherlands (sampled between 2003 and 2007) within the East Asia clade, *ermB* was present in all but the most recently sampled isolate.

Having determined from the sequencing data whether genes encoding PVL (*lukS/F*) were present or absent in our 120 isolates, we found that there was a significant difference in distribution between the USA and East Asia clades. Although isolates in the East Asia clade were generally more likely to carry PVL genes (61 % of the East Asia clade and 6 % of the USA clade were PVL gene-positive), a difference in PVL gene carriage was also observed within the two major subclades of the East Asia clade (Fig. 4), where 4 % (1/26 isolates) and 93 % (53/57 isolates), respectively, were PVL gene-positive. The split within the East Asia clade into one clade associated with the presence of PVL genes and the other associated with their absence corresponded closely with the second most basal split in the hierarchical clustering analysis of the whole accessory genome, which separated isolates in the East Asia clade into two clusters (Fig. 4; Additional file 1: Figure S10).

## Discussion

In this study we used whole genome analysis to shed light on the intercontinental transmission of *S. aureus* CC59 and determined the ancestral relationships between CC59 isolates from five countries around the world (Australia, the Netherlands, Taiwan, the UK and the USA), resolving a long-standing mystery in the field. From the whole-genome sequencing data we described the distribution of genes encoding antibiotic resistance and bacterial virulence across the phylogeny to predict the phenotypic properties of the isolates.

Our core genome analysis supported the existence of two major clades, one originating in the USA and the other in East Asia. No evidence was obtained for the hypothesis, discussed previously in the literature [20] and based on the timing of case reports, that the ‘Taiwan clone’ of ST59 previously identified in the literature emerged as a descendent of USA1000. Rather, the East Asia clade (which includes strains from Taiwan) and the USA clade of ST59 appear to have emerged independently at similar times, in the 1960–1970s. A literature survey of published ST59 isolates (as of 29 May 2015) indicates that the earliest ST59 isolates reported are from 1994 in Taiwan and 1995 in the USA and that ST59 has been found in both countries simultaneously through to the present decade. Although we could not determine which clade originated first, the Bayesian skyride plot indicated that the expansion of the USA clade could have preceded the expansion of the East Asia clade. The expansion of the East Asia clade could speculatively relate to temporal differences in antibiotic use, for example the introduction of a new drug, and/or acquisition of an antibiotic resistance determinant. Of potential note, the CC59 isolate from Taiwan which joins at the most basal node of the East Asia clade lacked a number of resistance genes, including *catA*, e*rmB*, *ant(6)-Ia* and *aph(3)-III*, which were present in the majority of isolates from Taiwan.

Our finding of distinct subclades within the East Asia clade in terms of core genome, in addition to a distinct accessory genome profile including differences in PVL carriage, is consistent with reports in the literature based on laboratory typing and lower-resolution molecular methods which indicate that ST59 isolates from Taiwan belong to two clones. In particular, previous studies have speculated upon the existence of a ‘Taiwan clone’ (PVL-positive, SCC*mec* type V_T_) and an ‘Asian-Pacific clone’ (PVL-negative, SCC*mec* type IV) of ST59 [8, 12]. It is likely that our two major subclades within the East Asia clade, both of which contain isolates from Taiwan, correspond to these two clones.

Phylogenetic discrete-trait mapping methods provide a quantitative, hypothesis-driven framework for identifying sink and source populations for infection, in terms of geographic region, host populations, or species (e.g. [25–27]). Our results demonstrated the repeated transfer of CC59 between countries and continents, emphasising the ability of CA-MRSA strains to cross borders and highlighting the need for greater worldwide surveillance as a matter of global health importance. We identified the USA and East Asia as source populations for CC59, consistent with epidemiological inferences in the literature [19, 20], and Europe and Australia as sink populations. Hierarchical clustering analysis based upon the presence or absence of accessory genes indicated that isolates which belonged to the same major clade (USA or East Asia) in the core genome phylogeny also had more similar accessory gene content, similar to a previous observation for pneumococci [28].

The 120 isolates in our study were chosen from global CC59 samples to maximise spatiotemporal diversity. The high levels of consistency between results for the full dataset and the subsampled runs indicated that our BEAST analyses were not unduly influenced by uneven sampling (e.g. with regard to date) within the countries from which isolates were sequenced. We noted some sensitivity of the Bayesian skyline plots to sampling at later sampling times, consistent with previous reports [29]. Bayes factor support for links between countries, which has been suggested to be influenced by rare events [30], was also observed to be sensitive to sampling. The USA isolates in our study were predominantly commensal methicillin-sensitive *S. aureus* (MSSA) whereas a large number of the Taiwan isolates were from invasive MRSA infections (Additional file 1: Table S1); it is not clear whether this reflects sampling heterogeneity or genuine biological differences in the ability of ST59 strains in the USA and Taiwan to cause human infection. Although many of the USA isolates were from the American Midwest, they were diverse and strains from Western USA, Southwestern USA and USA1000 reference strains (NARSA-483 and NARSA-676) from the Northeast were interspersed amongst them in the core genome phylogeny. Future phylogenomics studies could investigate the spread of ST59 within the USA in more detail, as well as within East Asia; for example, mainland China has contributed a substantial number of isolates to world reporting since the mid-2000s.

As demonstrated in our study, CA-MRSA spreads frequently at the intercontinental level and thus populations are exposed to different strains. However, the dominance of *S. aureus* clones is known to vary within and between continents in the community setting. For example, whilst ST59 is the most prevalent CA-MRSA clone in Taiwan, in some other parts of Asia different multi locus sequence types, including ST30, are known to predominate (see [9, 31] for discussion). Furthermore, the ST8 USA300 clone has become the dominant CA-MRSA clone in North America but is less successful in other parts of the world [6]. It has been suggested that international travel could play a significant role in the replacement of endemic *S. aureus* strains with those of greater fitness [32]. However, the mechanisms through which community-associated *S. aureus* strains become, or remain, dominant within a geographical niche are yet to be elucidated. A recent study of healthcare-associated *S. aureus* infections concluded that clinical practice, antibiotic usage and competition between bacterial clones were important in determining nosocomial bacterial population dynamics [33]. Enhanced understanding of the factors affecting clonal competition could facilitate more accurate predictions about the spread of emerging bacterial clones and their potential to compete with resident strains [34].

We found that ST59 isolates from Taiwan possessed a greater number of genes associated with antibiotic resistance than isolates from the USA and were predicted to be resistant to a larger number of antibiotic classes. Antibiotic usage can lead to resistance at the population level [35] and high usage levels, including non-prescription use [36], are thought to contribute to the prevalence of antibiotic resistance in Taiwan being amongst the highest in the world [37]. Macrolide and beta-lactam resistance genes (*ermB* and *mecA*, respectively) were associated with the East Asia ST59 clade in our study and high levels of resistance to these drugs in Taiwan have also been reported for another coloniser of the nasopharynx, *Streptococcus pneumoniae* [38–40]. The chloramphenicol resistance gene *catA* was also associated with the East Asia clade; indeed, chloramphenicol resistance has been reported amongst the earliest Taiwanese ST59 strains [16, 41] and at high levels in other bacteria in this region (e.g. [42–44]). Although not widely prescribed orally in humans today due to high toxicity levels, and banned in food animals in Taiwan since 2003, concerns have been voiced about illegal chloramphenicol use in aquaculture in parts of Southeast Asia [45] and are the basis for the EU’s rejection of some aquaculture products from Southeast Asia [46]. In our study we identified the spread of resistant strains into Europe that could have emerged due to antibiotic selection in a different location (Taiwan/East Asia) and this should be addressed in greater detail by enhanced global surveillance and future phylogenetic studies of larger datasets.

We hypothesise that the lack of transfer observed between ST59 from Taiwan and the USA in our phylogenetic analysis could relate to differences in antibiotic resistance and usage in these regions. It is possible that the USA strains are too susceptible to antibiotics to become established in Taiwan, where antibiotic usage is higher. Resistance mechanisms may be associated with a fitness cost which manifests as a reduced bacterial growth rate in the absence of antibiotic pressure [47, 48], and fitness costs have been demonstrated for specific classes of antibiotic in *S. aureus* [48]. Increased carriage of resistance determinants might, therefore, lead the Taiwanese strains to be outcompeted by USA ST59 or other *S. aureus* strains in regions with lower levels of antibiotic usage.

Although one might expect Europe to be more similar to the USA than Taiwan in terms of antibiotic use, we observed sub-clades of ST59 strains from Europe in both the USA and East Asia clades, suggestive of the capacity for onward transmission in Europe of strains from both of these locations. In considering why ST59 from both clades can spread in Europe, we may need to consider competition dynamics with the predominant community-associated strains there such as ST80. Future studies comparing the gene content of well-sampled groups of global isolates from multiple clonal complexes of CA-MRSA, including CC59, USA300 and ST80, could prove useful in identifying the basis for the dominance of particular clones in different geographical niches.

For community-associated *S. aureus*, direct competition between strains is likely to take place in the nasopharynx and might also involve competition with other residents of that niche, such as *S. pneumoniae* [49], which could be further investigated through laboratory experiments. Indeed, it is possible that the most biologically relevant competition that CA-MRSA strains face is with the rest of the host microbiome, rather than directly with other *S. aureus* strains. *S. aureus* is found amongst the gut microbiota (discussed in [50]) as well as the skin microbiota (discussed in [51]) and microbiome content has been found to vary at the intercontinental level, including between westernised and non-westernised countries in the gut [52] and between the USA and Asia (China) on skin sites [53]. Furthermore, host genetic variation, including between American and Asian populations, has recently been associated with differences in microbiome content at a number of body sites [54]. We therefore postulate that combining host ethnicity and microbiome data (which were not available for the strains we examined) could provide some explanation as to the geographical exclusion observed between ST59 strains from the USA and Taiwan in this study.

## Conclusions

We traced the emergence and dissemination of two major clades of *S. aureus* ST59, one in the USA and one in East Asia and, contrary to previous suggestions, did not find evidence for the Taiwan ST59 clone having emerged from the USA. Our quantitative analysis of the global circulation of ST59 identified East Asia and the USA as separate source populations and Europe and Australia as sink populations, revealing pathways for the global spread and frequent intercontinental transmission of ST59 CA-MRSA. We also shed light on important genomic differences between ST59 isolates from the USA and Taiwan. Of note, the lack of direct exchange of ST59 strains between the USA and Taiwan is striking and may in part reflect their remarkably different profiles of mobile genetic elements. In particular, ST59 strains from Taiwan possess a much larger number of antibiotic resistance genes than the USA strains, an observation that is consistent with distinct cultures of antibiotic usage in the different regions. Taken together, these findings highlight the critical requirement for a global strategy to control the emergence and dissemination of antibiotic-resistant clones.

## Methods

### CC59 isolates

We obtained CC59 isolates from laboratories around the world (in the UK, Netherlands, USA, Taiwan and Australia) to reflect the global distribution of countries where CC59 samples have been identified and are available (Table 1; Additional file 1: Figure S1 and Figure S2). The date (year) and country in which each sample was taken were also obtained. DNA was extracted using a commercial kit.

### Sequencing, assembly and alignment

Whole-genome sequencing, using the Illumina Mi-Seq platform, was performed by Edinburgh Genomics at the Roslin Institute, Edinburgh, United Kingdom. Short reads from the Illumina sequencing were aligned by mapping to an ST59 reference sequence (GenBank accession CP003166) using the Burrows–Wheeler Aligner (BWA) with the Smith-Waterman algorithm disabled [55] as outlined previously [56]. A core genome alignment was created from the consensus sequences, with the core genome defined as nucleotide sites shared by all sequences (alignment columns that did not contain a gap character or unknown nucleotide identity). Sites were excluded if there was an ‘N’ base-call or a gap at that position for any sequence. (See Additional file 2 for list of included sites, relative to the reference genome.) Our core genome alignment was 2,442,017 base pairs in length, of which 7061 sites were variable.

### In silico multi-locus sequence typing

SRST2 [57] was used to align short reads to reference alleles for the seven housekeeping loci used to define multi-locus sequence types to determine whether isolates belonged to CC59. Multi-locus sequence typing analysis revealed 101 isolates (84 % of the 120 isolates) to be ST59, with other sequence types (ST87, ST338, ST375, ST952) which are single locus variants of ST59 also represented in the dataset at much lower levels (0.8, 5, 1.6 and 0.8 %, respectively). Nine isolates, all of which had been identified as ST59 in the laboratory, were more difficult to assign multi-locus sequence types to using the in silico method: two matched the ST59 allelic profile except for containing a single-nucleotide polymorphism (SNP) at one locus; five matched the ST59 profile except for some uncertainty (e.g. low coverage) at one or more loci, and for two isolates the multi-locus sequence types could not be determined. ST59 isolates were found in all of the five countries studied. Subsequent maximum likelihood phylogenetic analysis of the core genome did not reveal any outlying sequences, including amongst isolates with the novel or undefined sequence types.

### Recombination detection

The core genome alignment was screened for recombination using the BratNextGen [58] software, three different methods in RDP v4.19 [59] and a single breakpoint analysis [60] in the HyPhy software [61]. Putative recombinant regions were removed prior to phylogenetic analysis (the region from sites 192166–192887 inclusive was removed from the core genome), although this did not affect the phylogenetic clustering into major clades.

### Preliminary phylogenetic analysis of CC59

Maximum likelihood phylogenetic analyses were carried out using neighbour-joining methods in MEGA v5.2 [62], maximum likelihood in RAxML (Linux version v7.2.8) [63] and PhyML v3.0 [64]. The GTR nucleotide substitution model was used, with gamma-distributed rate heterogeneity across sites and 1000 bootstrap replicates performed. The resulting phylogenetic trees were rooted at the midpoint between the two most divergent taxa in the tree. The temporal signal present in the dataset was investigated through a plot of root-to-tip distance against the year of sampling in TempEst [65] and a linear regression was carried out in R to assess the significance of this relationship. An overall pattern of increasing root-to-tip distance with sampling date was observed, indicating that diversity had accumulated in a clock-like fashion over time (Additional file 1: Figure S11).

### Phylogeographic analysis of the global dispersal of CC59

A Bayesian phylogeographic analysis of the global dissemination of ST59 on an explicit time-scale (labelling sequences by their year or sampling) was carried out using the BEAST software [66]. A relaxed (uncorrelated lognormal) molecular clock model and a relaxed demographic prior (Bayesian skyride) were selected on the basis of Bayes factor testing between different molecular clock models and coalescent tree-priors. An HKY85 [67] model of nucleotide substitution was selected, with gamma-distributed rate variation and a proportion of invariant sites.

Sequences were labelled according to the country of sampling and the dissemination of CC59 between countries was modelled in BEAST as a continuous-time Markov chain [68]. The Bayesian stochastic search variable selection (BSSVS) procedure was used to identify links between countries which were required in a symmetric diffusion model to most parsimoniously explain the observed pattern of sampling locations at the tips of the phylogeny (using Bayes factor cutoffs of (i) 20 and (ii) 3). In order to quantify transitions in and out of different countries, a Markov jumps analysis [69–72] was implemented under an asymmetric diffusion model to count the number of jumps across the phylogeny samples from one country to another. The Markov jumps log files were processed in R to investigate the number CC59 jumps in and out of different countries over time.

### Subsampling analysis

To assess the robustness of our phylogeographic inference to biases in the dataset which could occur through uneven sampling, we conducted phylogeographic analyses on ten subsampled datasets. Subsampling of the original dataset with respect to date and country of sampling was performed to thin the original dataset of 120 sequences in one of two ways: (i) five datasets of 50 sequences (ten per country, randomly sampled across years) were obtained; (ii) five datasets of 50 sequences were sampled as in (i) but omitting sequences sampled before 2002 to remove potential bias arising from uneven distributions of available sequences across countries before this point. Markov jumps and BSSVS analyses were conducted on the subsampled datasets using the same approach as described for the full dataset.

### De novo assembly and accessory genome analysis

De novo assembly of genomes for the 120 CC59 isolates described in the phylogenetic analysis was carried out using SPAdes [73] and the assemblies were annotated using Prokka [74]. The NUCmer software [75] was used to create a reference pangenome by stepwise addition of new genes found in each of the CC59 genomes. The bi-directional best hits (BDBH) method in *get_homologues* [76] was used to compare all CC59 assemblies against the reference pangenome. The presence or absence of all genes in the reference pangenome was determined for each of the 120 ST59 isolates based upon chosen similarity and coverage thresholds as well as paralogous genes within genomes. A range of thresholds were considered (default settings were compared with similarity values of 70, 80 and 90 %, all with coverage = 75 % and E-value = 1e-05). Tabulated pan-genome matrices were created using the *compare_clusters.pl* script downloaded as part of the *get_homologues* package.

The resulting pangenome matrix was coded to binary, with 0 representing absence and entries greater than 0 (i.e. including groups of paralogous genes) coded to 1 to signify the presence of a gene. Columns (corresponding to genes) with less than 10 % variability with respect to gene presence or absence across the CC59 genomes were removed and heat maps of gene presence and absence were plotted using *heatmap.2* in the *gplots* package in R. Hierarchical clustering was used to group the isolates according to similarity in the distribution of accessory genes.

### In silico detection of antibiotic resistance genes and SNPs

SRST2 [57] was used to detect genes known to be antibiotic resistance determinants by mapping the short read data against reference gene sequences from the ARG-ANNOT database [77]. Association of resistance genes with CC59 isolates from Taiwan or the USA was tested using Fisher’s exact tests with a Bonferroni multiple testing correction. BLAST was used to search for SNPs in the *gryA*, *grlA* and *grlB* genes associated with resistance to ciprofloxacin, using a list of SNPs from [78]. Presence or absence of PVL was determined from the short read data using the procedure described in [25]. Output used to generate Fig. 4 can be found in Additional file 3.

In addition, Fisher’s exact test was used to identify genes identified from the pangenome analysis whose presence or absence differed significantly between (i) the ‘Taiwan’ or ‘USA’ clades or (ii) isolates from Taiwan or the USA (i.e. excluding genomes from other countries). The direction of association was also identified (see Additional file 4 for significant genes). A Bonferroni correction based upon the number of column patterns (with each column representing the distribution of a particular gene across the 120 isolates) for multiple testing was applied, which is conservative but would reduce the chance of genes being identified spuriously as associated with either clade. Gene name information was taken from the Prokka annotations and from additional BLAST searches of protein sequences against the TrEMBL and SWISS-PROT databases for sequences labelled ‘hypothetical protein’ by Prokka. A literature search was conducted to identify members of the set of significant genes which have been found to be associated with antibiotic resistance, bacterial virulence or pathogenesis or bacterial competition.

## Abbreviations

BSSVS, Bayesian stochastic search variable selection; CA-MRSA, community-associated MRSA; CC, clonal complex; HPD, highest posterior density; MRSA, methicillin-resistant *Staphylococcus aureus*; PP, posterior probability; PVL, Panton-Valentine leukocidin; SNP, single nucleotide polymorphism; ST, (multi locus) sequence type

## Additional files

Additional file 1: Supplementary figures and table of isolates. **Figure S1.** Global distribution of reported ST59 isolates and sequencing in our study. **Figure S2.** Spatiotemporal distribution of CC59 sequences in this study. **Figure S3.** Maximum likelihood phylogeny of global CC59 sequences from humans. **Figure S4.** BEAST maximum clade credibility tree of global human CC59 sequences from humans. **Figure S5.** Posterior probabilities for ancestral location of USA-associated clade in subsampled runs. **Figure S6.** Posterior probabilities for ancestral location of East Asia-associated clade in subsampled runs. **Figure S7.** Bayes factors indicating support for links between countries in a symmetric BSSVS phylogeography analysis. **Figure S8.** Skyride plots for subsampled runs. **Figure S9.** Heatmaps of median number of transitions between countries for subsampled runs. **Figure S10.** Presence or absence of accessory genes amongst global CC59 isolates. **Figure S11.** Root-to-tip distance plot for RAxML phylogeny of *S. aureus* CC59 sequences. **Table S1.** 120 ST59 isolates included in phylogenetic analysis. (DOCX 1792 kb) Additional file 2: List of core genome sites included in our analysis. Site numbers are relative to the published ST59 genome used as a reference for mapping short reads (GenBank accession CP003166). (TXT 20378 kb) Additional file 3: Output from in silico antibiotic resistance testing. Genes associated with antibiotic resistance were detected using SRST2. Gene presence is denoted by the name of the gene (*asterisks* indicates at least one mismatched SNP or indel and a *question mark* indicates some low-depth bases, as described on the SRST2 website: https://github.com/katholt/srst2). Gene absence is denoted by a *hyphen*. The amino acid residue (IUPAC single letter code) is given for *gyrA*, *grlA* and *grlB* sites associated with fluoroquinolone resistance. Presence or absence of PVL based on mapping of short reads to *lukF-PV* and *lukS-PV* reference sequences is also reported. (XLSX 16 kb) Additional file 4: Genes associated with either USA or East Asia clade. Genes whose presence differed significantly between the USA and East Asia clades are listed. We also report the proportion of isolates within the USA and East Asia clades for which each gene was present and note those that share the same pattern of presence or absence across the isolates (identified by having the same ‘column pattern’). Results for genes whose presence differed significantly between the USA and Taiwan isolates (as opposed to the USA and East Asia clades) are presented in a separate worksheet. (XLSX 26 kb)
