# Supplementary material for: Identification of source and sink populations for the emergence and global spread of the East-Asia clone of community-associated MRSA
Source: Genome Biol. 2016 Jul 26;17:160. doi: 10.1186/s13059-016-1022-0 (PMC4962458; doi:10.1186/s13059-016-1022-0)
Supplement: Additional file 1: — Supplementary figures and table of isolates. Figure S1. Global distribution of reported ST59 isolates and sequencing in our study. Figure S2. Spatiotemporal distribution of CC59 sequences in this study. Figure S3. Maximum likelihood phylogeny of global CC59 sequences from humans. Figure S4. BEAST maximum clade credibility tree of global human CC59 sequences from humans. Figure S5. Posterior probabilities for ancestral location of USA-associated clade in subsampled runs. Figure S6. Posterior probabilities for ancestral location of East Asia-associated clade in subsampled runs. Figure S7. Bayes factors indicating support for links between countries in a symmetric BSSVS phylogeography analysis. Figure S8. Skyride plots for subsampled runs. Figure S9. Heatmaps of median number of transitions between countries for subsampled runs. Figure S10. Presence or absence of accessory genes amongst global CC59 isolates. Figure S11. Root-to-tip distance plot for RAxML phylogeny of S. aureus CC59 sequences. Table S1. 120 ST59 isolates included in phylogenetic analysis. (DOCX 1792 kb) [file 13059_2016_1022_MOESM1_ESM.docx]

**Supplementary figures and table**

**
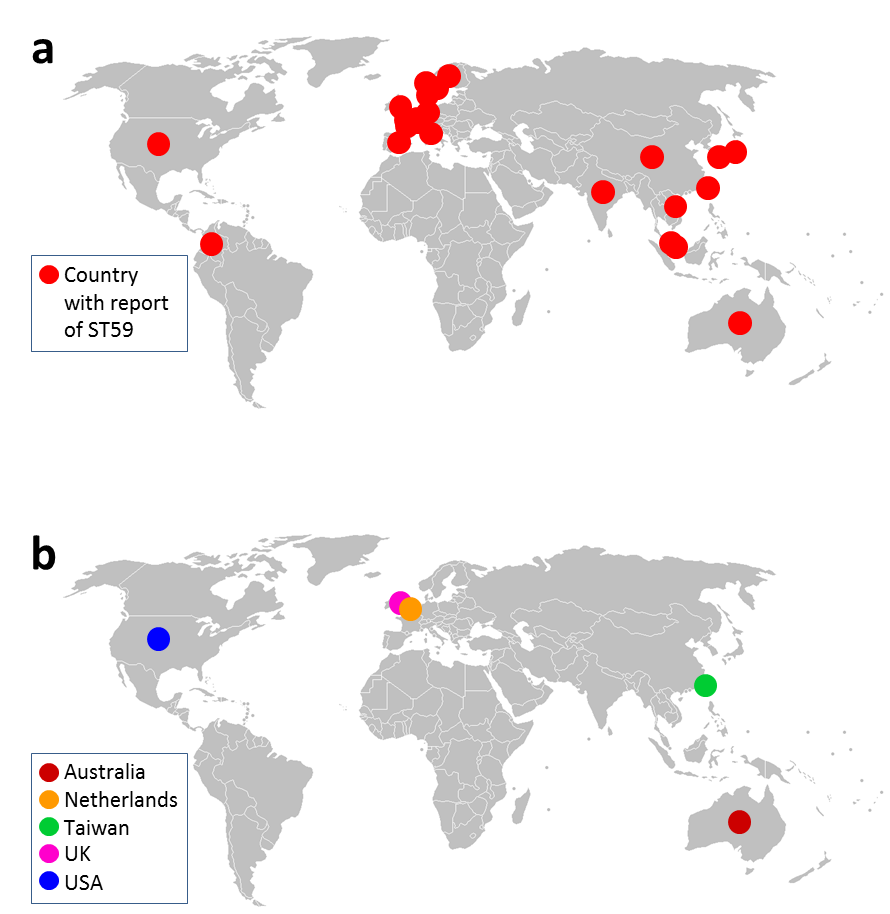
**

**Figure S1: Global distribution of reported ST59 isolates and sequencing in our study.** (a) ST59 cases have been reported in many countries globally, including Taiwan, Australia, the Netherlands, the UK the USA, Belgium, China, Columbia, Denmark, Finland, France, Germany, India, Italy, Japan, Luxembourg, Malaysia, Norway, Singapore, South Korea (from cattle carcass), Spain, Sweden and Vietnam (information collated from a survey of the literature – see Main Text). (b) We obtained CC59 isolates from laboratories around the world to reflect the global distribution of infection. These isolates were then subjected to whole genome sequencing and subsequent phylogeographic analysis to investigate the dissemination of CC59 around the world.

**
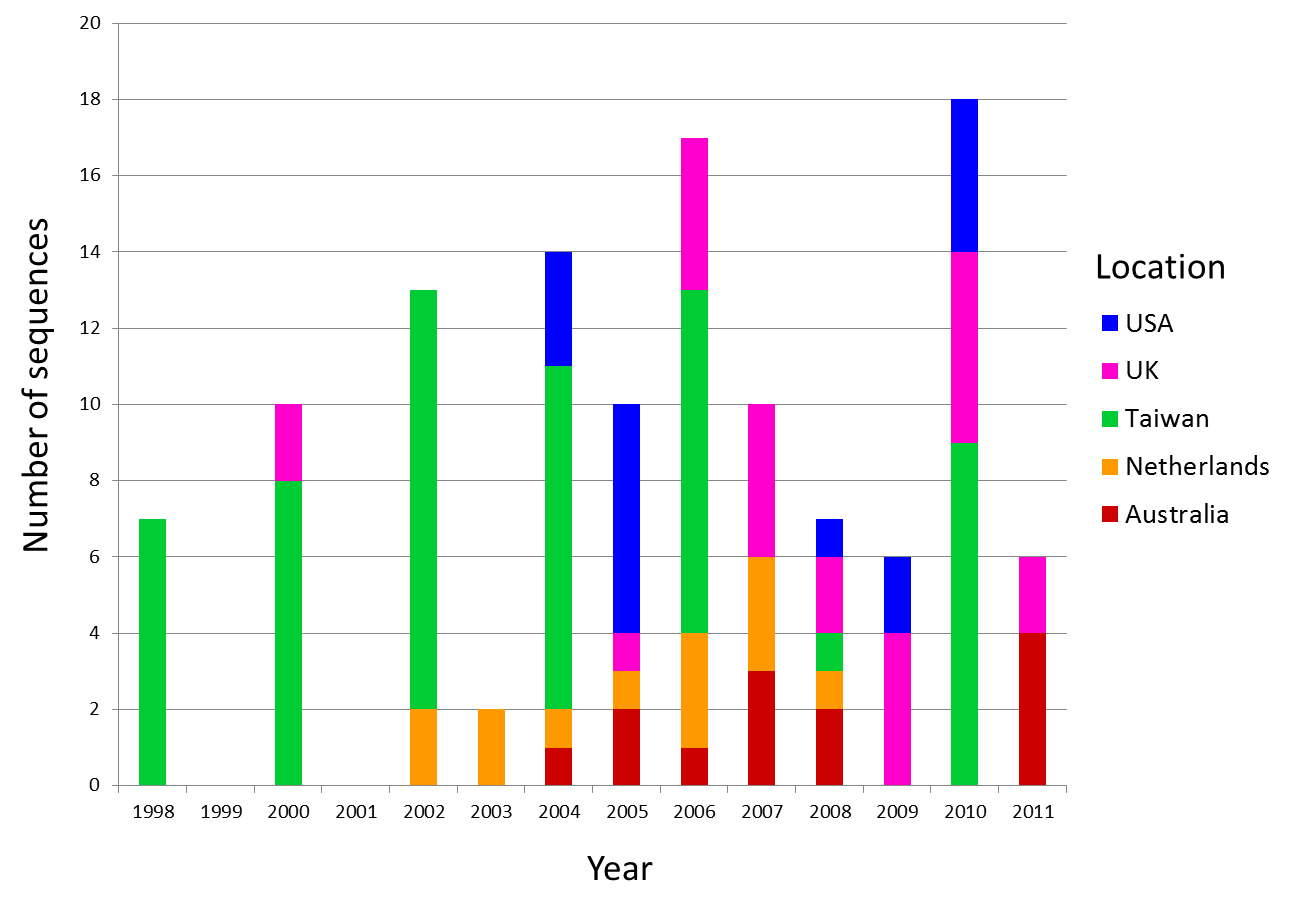
**

**Figure S2: Spatiotemporal distribution of CC59 sequences in this study.** CC59 isolates were obtained from laboratories around the world and subjected to whole genome sequencing. Countries were chosen according to the availability of ST59 isolates from sending laboratories and to reflect the global distribution of CC59. For each country, isolates for sequencing were chosen to maximise temporal diversity. 120 CC59 whole genomes were available for phylogenetic analysis after quality control.


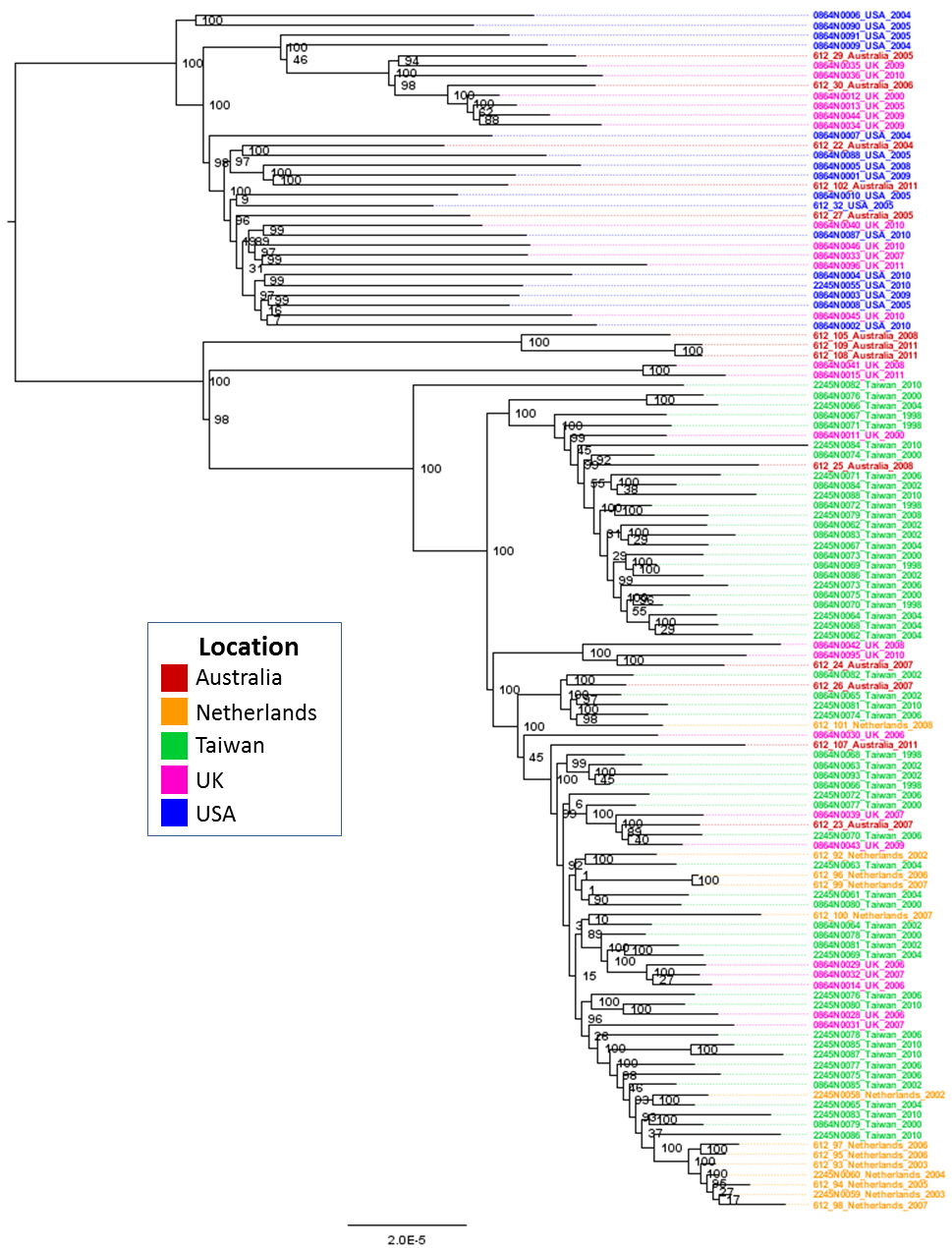


**Figure S3: Maximum likelihood phylogeny of global CC59 sequences from humans.** The tree was constructed using the RAxML software. Sequence labels are coloured according to the country from which sequences were sampled. Bootstrap values based upon 1000 bootstrap replicates are shown and expressed as percentages. The tree has been midpoint rooted. The scale is in units of substitutions per site.

**
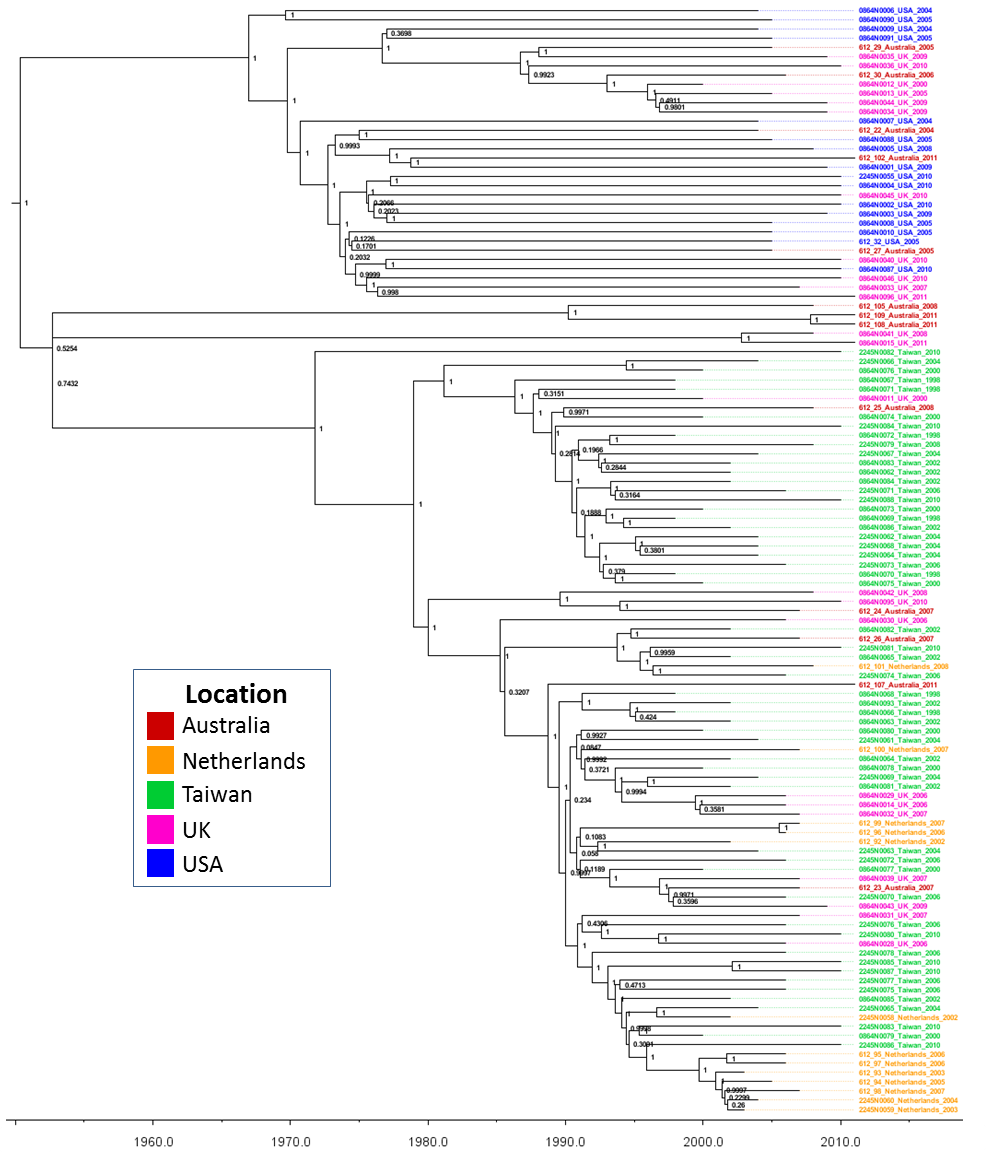
**

**Figure S4: BEAST maximum clade credibility tree of global human CC59 sequences from humans.** Sequence labels are coloured according to the country from which sequences were sampled. Nodes are labelled with posterior probability values. The tree is shown on an explicit timescale. There was considerable difficulty in placing a group of 3 sequences from Australia (612_105, 612_108 and 612_109) and 2 sequences from the UK (0864N0015 and 0864N0041), as indicated by low posterior support for their position in the tree; however, the major clustering into East Asia- and USA-associated clades was not affected by their presence and remained the same when the phylogenetic analysis was re-run with the 5 sequences excluded.


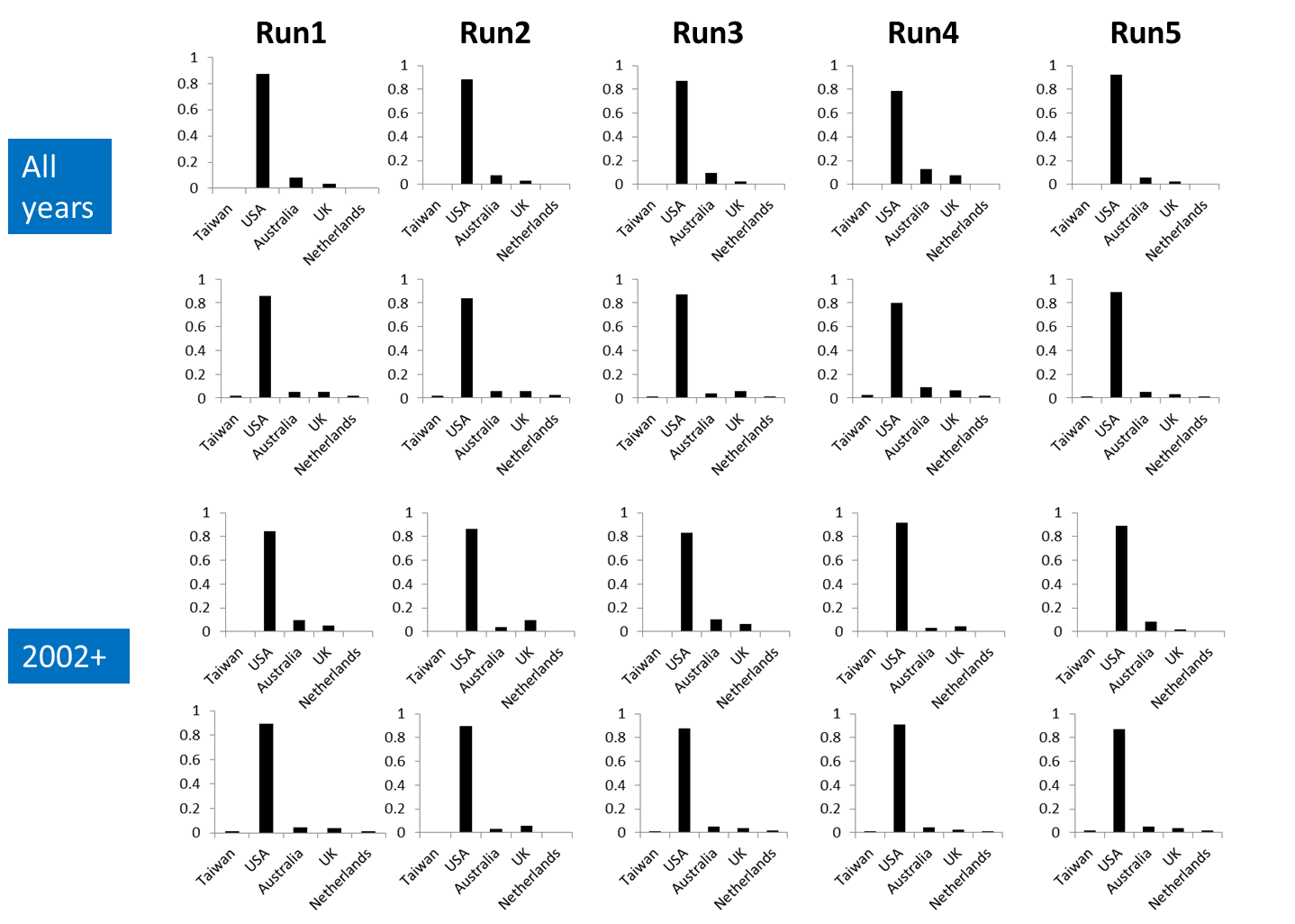


**Figure S5: Posterior probabilities for ancestral location of USA-associated clade in subsampled runs.** The CC59 sequence dataset was down-sampled 5 times (denoted Run1-Run5) from 120 to 50 sequences for (i) the full dataset and (ii) only sequences from 2002 onwards, as described in the main text. Two BEAST phylogeography replicates were performed on each subsampled dataset, and the posterior probability of the ancestral location for the clade containing USA sequences was recorded. In support of our results from the full dataset, the USA was inferred to be the ancestral location of the clade containing sequences from the USA in all runs and replicates. Plots show the posterior probability of the ancestral location of the clade being in different countries (x-axis = country; y-axis = posterior probability). For all subsampled datasets, the 95% HPD intervals for the emergence date of the USA clade overlapped with those for the full dataset.


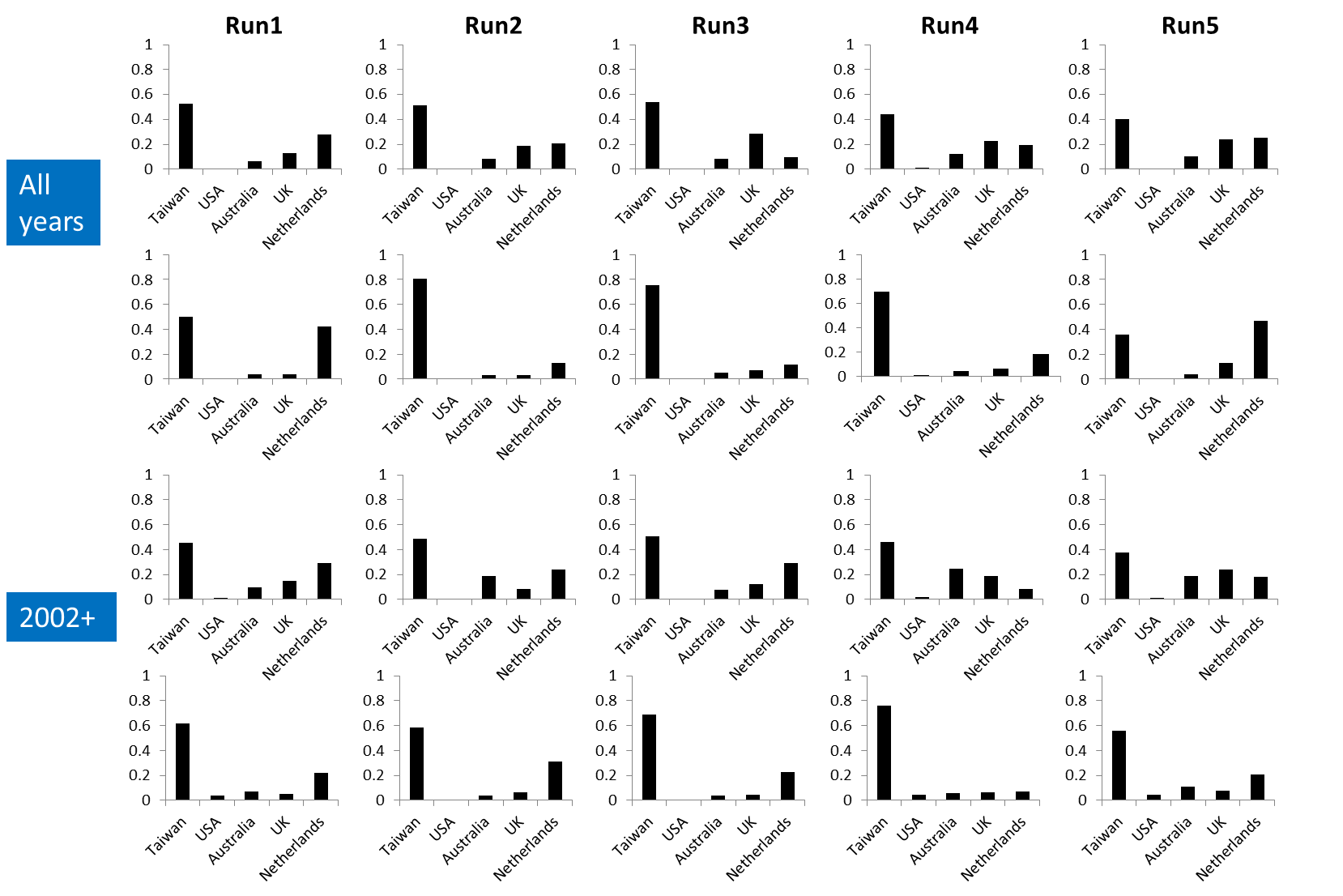


**Figure S6: Posterior probabilities for ancestral location of East Asia-associated clade in subsampled runs.** The CC59 sequence dataset was down-sampled 5 times (denoted Run1-Run5) from 120 to 50 sequences for (i) the full dataset and (ii) only sequences from 2002 onwards, as described in the main text. Two BEAST phylogeography replicates were performed on each subsampled dataset, and the posterior probability of the ancestral location for the major clade containing sequences from Taiwan (the ‘East Asia’ clade) was recorded. The highest posterior probability for the ancestral location was inferred to be Taiwan in 19 out of 20 runs and replicates, consistent with the results from the full dataset. Plots show the posterior probability of the ancestral location of the clade being in different countries (x-axis = country; y-axis = posterior probability). For all but one subsampled dataset, the 95% HPD intervals for the emergence date of the East Asia clade overlapped with those for the full dataset.


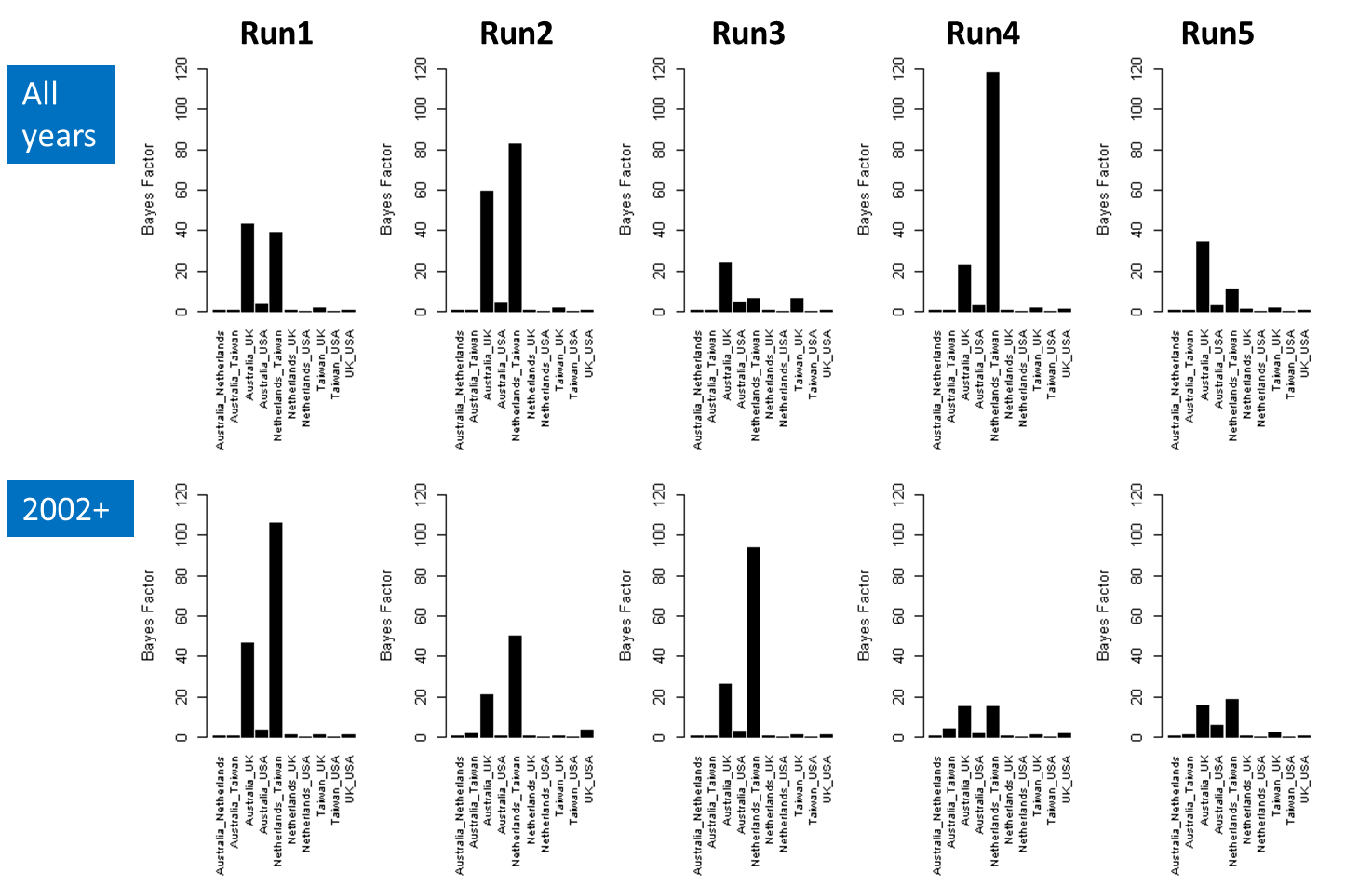


**Figure S7: Bayes factors indicating support for links between countries in a symmetric BSSVS phylogeography analysis.** The CC59 sequence dataset was down-sampled 5 times (denoted Run1-Run5) from 120 to 50 sequences for (i) the full dataset and (ii) only sequences from 2002 onwards, as described in the main text. Bayesian stochastic search variable selection (BSSVS) was used to assess support for symmetric links between pairs of countries in the simplest model required to explain the distribution of countries at the tips of the tree. Plots show Bayes factors indicating support for links between pair of countries. Although the two most strongly supported links between countries was consistent over replicates, variation between Bayes factor support for links between countries differed between subsampled runs, both in terms of the absolute values of the Bayes factors for particular links and in the relative sizes of the Bayes factors for different pairs of countries, suggesting that the BSSVS phylogeography method is particularly sensitive to sampling effects.


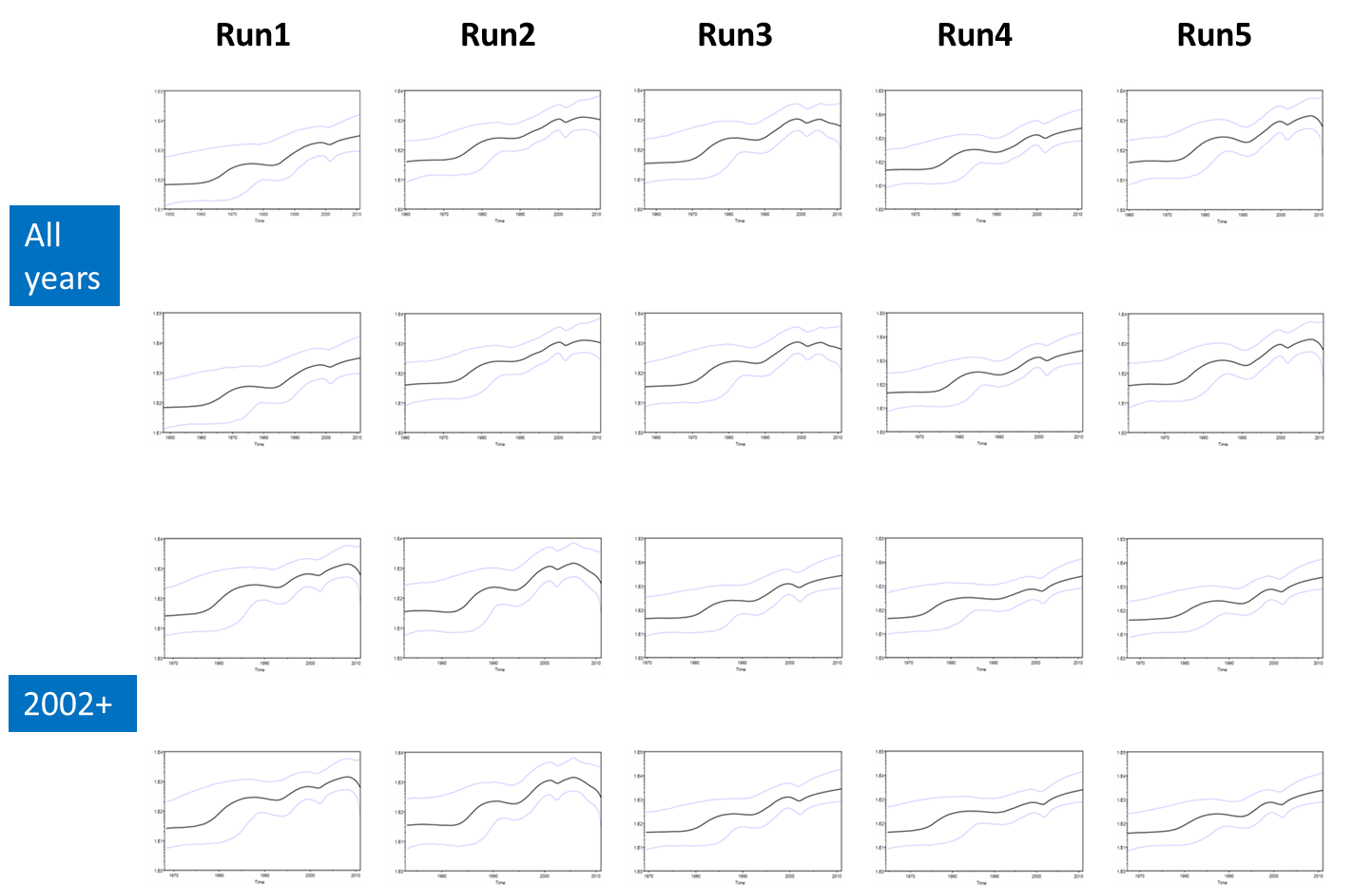


**Figure S8: Skyride plots for subsampled runs.** The CC59 sequence dataset was down-sampled 5 times (denoted Run1-Run5) from 120 to 50 sequences for (i) the full dataset and (ii) only sequences from 2002 onwards, as described in the main text. Two BEAST phylogeography replicates were performed on each dataset, and Bayesian skyride plots were reconstructed. Similar trends in the skyride plots can be observed across runs and replicates as in the full dataset, with peaks in relative genetic diversity corresponding to the expansion of the USA clade followed by expansion of the East Asia clade, although there is some variation in particular in the most recent past, consistent with the method being particularly sensitive to sampling effects at the youngest tips of the tree. Plots show relative genetic diversity (y-axis) over time (x-axis).

**
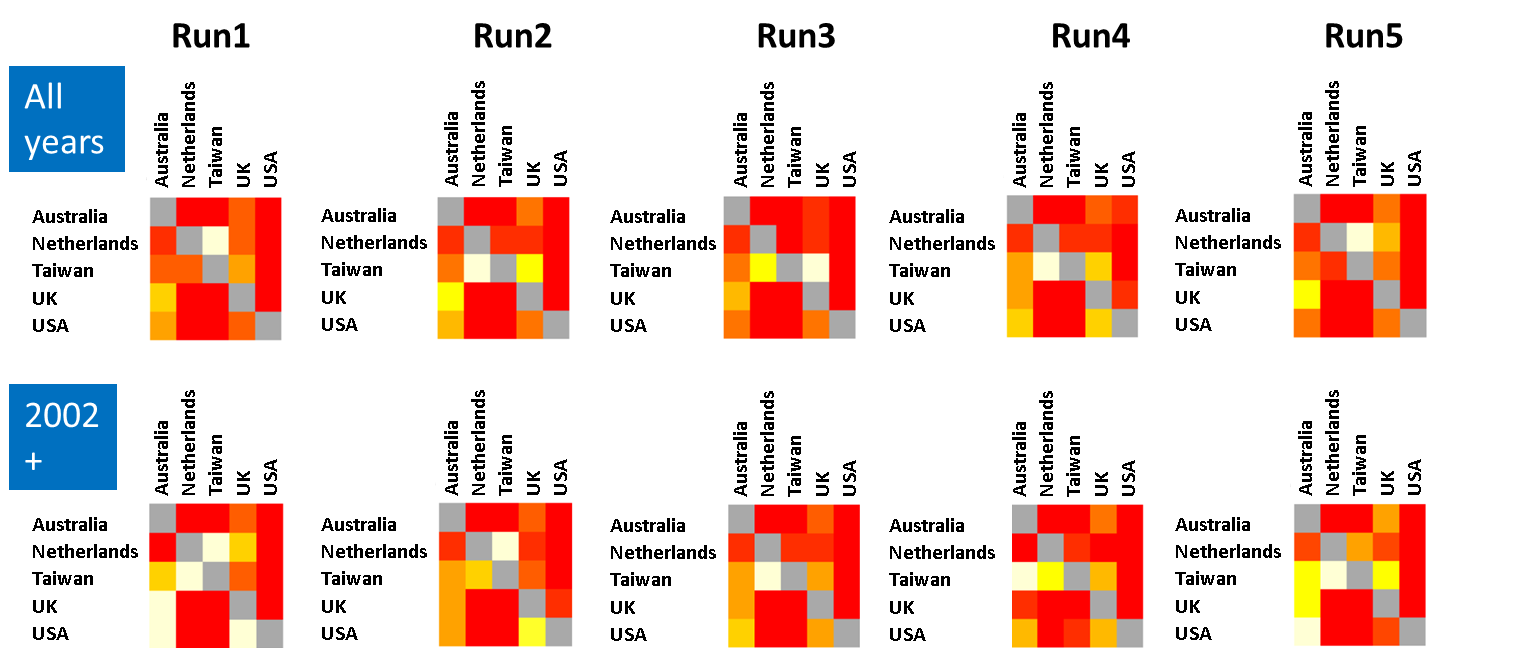
**

**Figure S9: Heatmaps of median number of transitions between countries for subsampled runs.** The CC59 sequence dataset was down-sampled 5 times (denoted Run1-Run5) from 120 to 50 sequences for (i) the full dataset and (ii) only sequences from 2002 onwards, as described in the main text. The median number of transitions from one country to another was calculated across posterior tree samples, and plotted as a heatmap, with lighter colours indicating a higher number of transitions. Similar trends in the pattern of transitions from one country to another can be observed across the subsampled runs, and are largely consistent with the results from the full dataset (see Figure 3 in Main Text).

**
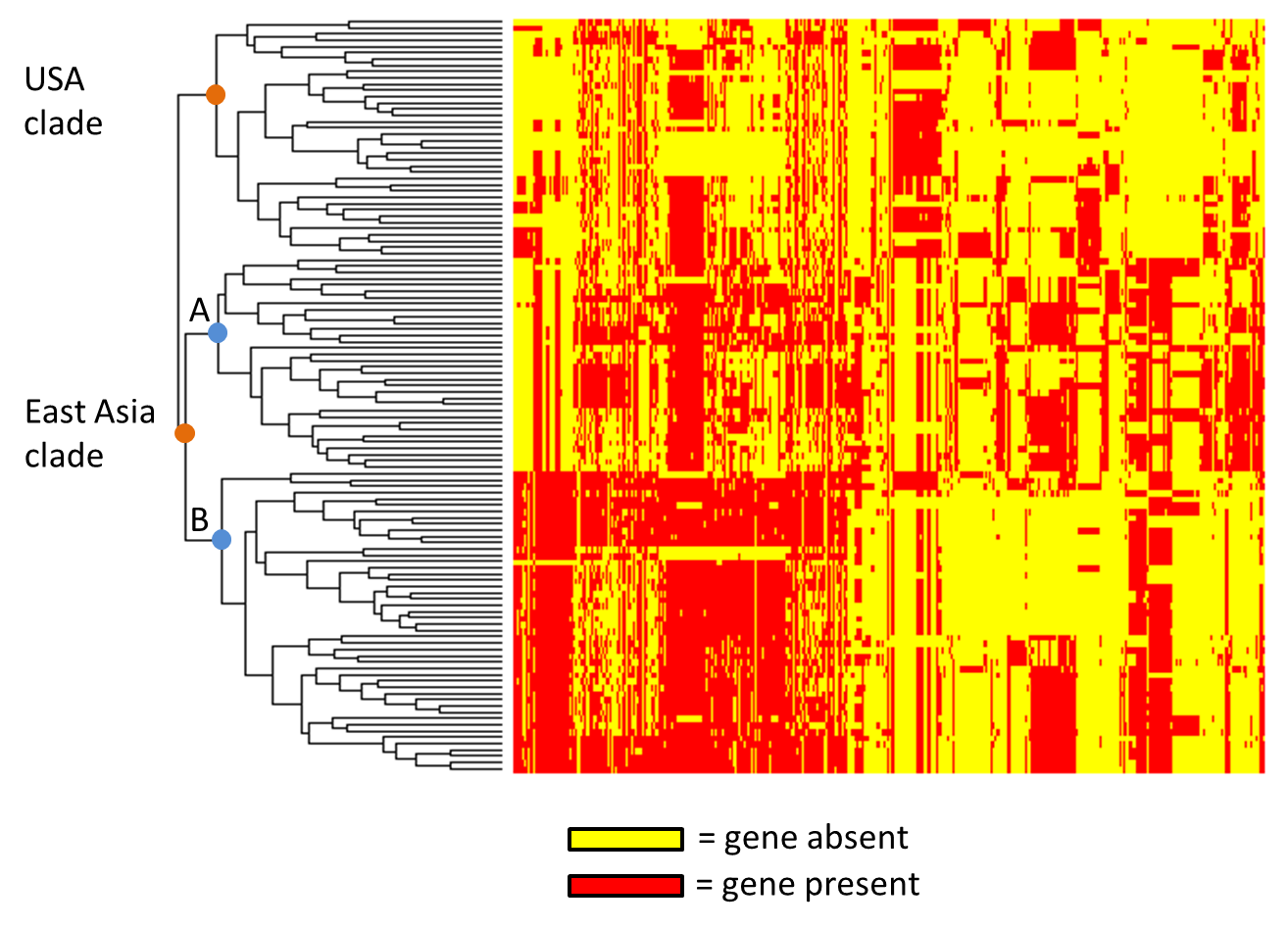
**

**Figure S10: Presence or absence of accessory genes amongst global CC59 isolates.** After performing a pan-genome analysis for the CC59 isolates, accessory genes (i.e. those not present in all isolates) were identified. Genes with at least 10% variability in terms of presence or absence are plotted here, with columns corresponding to genes and rows corresponding to individual CC59 isolates. Presence of a gene in an isolate is indicated by red, with absence of a gene indicated by yellow. Hierarchical clustering was performed to cluster the isolates based upon accessory genome content, and the major split corresponded to the USA and East Asia clades observed in the core genome phylogenetic analysis (orange dots). The second most basal split was within the East Asia clade (blue dots – subclades A and B). All isolates within East Asia subclade B were members of the PVL-positive subclade of the East Asia clade identified in Figure 4 of the main text.


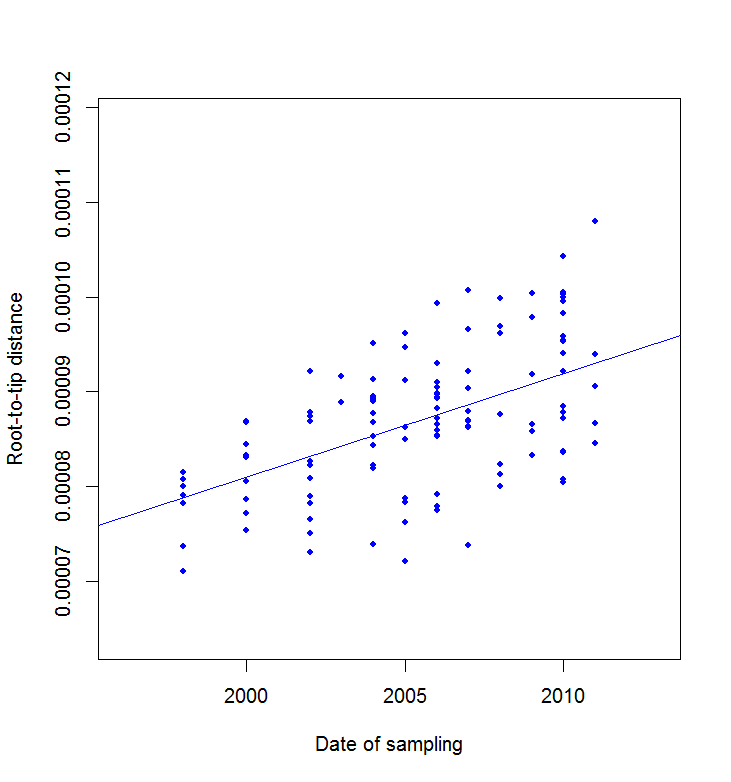


**Figure S11: Root-to-tip distance plot for RAxML phylogeny of *S. aureus* CC59 sequences.** The distances between the tips and the root of the tree (with the best-fitting root selected) were calculated using Path-O-Gen and plotted against the year of sampling.

| **Sample ID** | **Lab ID** | **Country** | **Year** | **ST  (*in silico*)** | **ST  (lab)** | **Sample type** |
| --- | --- | --- | --- | --- | --- | --- |
| 612_22 | WA MRSA-24 | Australia | 2004 | 87 | 87 | Leg pustule |
| 612_27 | WA MRSA-73 | Australia | 2005 | 59 | 59 | Nose |
| 612_29 | WA 05-17759 | Australia | 2005 | 59 | 59 | Wound swab |
| 612_30 | WA 06-18653 | Australia | 2006 | NF*? | 59 | Back |
| 612_24 | WA MRSA-55 - PVL pos | Australia | 2007 | 59 | 59 | Wound swab |
| 612_26 | WA MRSA-56 | Australia | 2007 | 59 | 59 | Abscess swab |
| 612_23 | WA MRSA-52 / Taiwan A | Australia | 2007 | 952 | 952 | Nose |
| 612_25 | WA MRSA-55 - PVL neg | Australia | 2008 | 59 | 59 | nose |
| 612_105 | PQ08001 | Australia | 2008 | 59? | 59 | Sputum |
| 612_107 | PQ11479 | Australia | 2011 | 59 | 59 | Wound Swab |
| 612_109 | PQ11915 | Australia | 2011 | 59 | 59 | Wound Swab |
| 612_108 | PQ11513 | Australia | 2011 | 59* | 59 | Skin Swab |
| 612_102 | PQ11851 | Australia | 2011 | 59? | 59 | Skin Swab |
| 2245N0058 | 1110200185 | Netherlands | 2002 | 59 | 59 | 0 |
| 612_92 | 1110200468 | Netherlands | 2002 | 59 | 59 | 0 |
| 2245N0059 | 1110300753 | Netherlands | 2003 | 59 | 59 | 0 |
| 612_93 | 1110300013 | Netherlands | 2003 | 59? | 59 | 0 |
| 2245N0060 | 1110400404 | Netherlands | 2004 | 59 | 59 | 0 |
| 612_94 | 1110502155 | Netherlands | 2005 | 59 | 59 | 0 |
| 612_95 | 1110600846 | Netherlands | 2006 | 59 | 59 | 0 |
| 612_96 | 1110601857 | Netherlands | 2006 | 59 | 59 | 0 |
| 612_97 | 1110602354 | Netherlands | 2006 | NF*? | 59 | 0 |
| 612_100 | 1110701209 | Netherlands | 2007 | 59 | 59 | 0 |
| 612_98 | 1110700216 | Netherlands | 2007 | 59 | 59 | 0 |
| 612_99 | 1110700667 | Netherlands | 2007 | 59? | 59 | 0 |
| 612_101 | 1110803234 | Netherlands | 2008 | 59 | 59 | 0 |
| 0864N0066 | 980201 | Taiwan | 1998 | 59 | 59 | Wound |
| 0864N0067 | 980587 | Taiwan | 1998 | 59 | 59 | 0 |
| 0864N0068 | 980951 | Taiwan | 1998 | 59 | 59 | 0 |
| 0864N0069 | 981916 | Taiwan | 1998 | 59 | 59 | Blood |
| 0864N0070 | 983045 | Taiwan | 1998 | 59 | 59 | Respiratory |
| 0864N0071 | 985009 | Taiwan | 1998 | 59 | 59 | 0 |
| 0864N0072 | 985741 | Taiwan | 1998 | 59 | 59 | Wound |
| 0864N0073 | 200146 | Taiwan | 2000 | 59 | 59 | Blood |
| 0864N0074 | 200345 | Taiwan | 2000 | 59 | 59 | Blood |
| 0864N0075 | 201254 | Taiwan | 2000 | 59 | 59 | Blood |
| 0864N0076 | 201325 | Taiwan | 2000 | 59 | 59 | Respiratory |
| 0864N0077 | 202463 | Taiwan | 2000 | 59 | 59 | Pus |
| 0864N0079 | 204164 | Taiwan | 2000 | 59 | 59 | Biopsy |
| 0864N0080 | 204482 | Taiwan | 2000 | 59 | 59 | Pus |
| 0864N0078 | 204097 | Taiwan | 2000 | 338 | 338 | Blood |
| 0864N0062 | 02L198 | Taiwan | 2002 | 59 | 59 | Blood |
| 0864N0063 | 02M013 | Taiwan | 2002 | 59 | 59 | Pus |
| 0864N0064 | 02M004 | Taiwan | 2002 | 59 | 59 | Pus |
| 0864N0065 | 02C103 | Taiwan | 2002 | 59 | 59 | Wound |
| 0864N0082 | 02K143 | Taiwan | 2002 | 59 | 59 | Pus |
| 0864N0083 | 02K218 | Taiwan | 2002 | 59 | 59 | Blood |
| 0864N0084 | 02L035 | Taiwan | 2002 | 59 | 59 | Wound |
| 0864N0085 | 02M071 | Taiwan | 2002 | 59 | 59 | Pus |
| 0864N0086 | 02M111 | Taiwan | 2002 | 59 | 59 | 0 |
| 0864N0093 | 02M039 | Taiwan | 2002 | 59 | 59 | Ear |
| 0864N0081 | 02J068 | Taiwan | 2002 | 338 | 338 | Fluid |
| 2245N0061 | 04G086 | Taiwan | 2004 | 59 | 59 | Wound/Pus |
| 2245N0062 | 04H173 | Taiwan | 2004 | 59 | 59 | Sputum |
| 2245N0063 | 04J010 | Taiwan | 2004 | 59 | 59 | Wound/Pus |
| 2245N0064 | 04N190 | Taiwan | 2004 | 59 | 59 | Pus |
| 2245N0065 | 04P067 | Taiwan | 2004 | 59 | 59 | Pus |
| 2245N0066 | 04Y102 | Taiwan | 2004 | 59 | 59 | Wound |
| 2245N0067 | 04Y183 | Taiwan | 2004 | 59 | 59 | Urine |
| 2245N0068 | 04Z180 | Taiwan | 2004 | 59 | 59 | Respiratory |
| 2245N0069 | 04J084 | Taiwan | 2004 | 338 | 338 | Wound/Pus |
| 2245N0070 | 06G065 | Taiwan | 2006 | 59 | 59 | Wound |
| 2245N0071 | 06H194 | Taiwan | 2006 | 59 | 59 | Sputum |
| 2245N0072 | 06L188 | Taiwan | 2006 | 59 | 59 | Wound/Pus |
| 2245N0073 | 06M065 | Taiwan | 2006 | 59 | 59 | 0 |
| 2245N0074 | 06S077 | Taiwan | 2006 | 59 | 59 | Pus |
| 2245N0075 | 06T005 | Taiwan | 2006 | 59 | 59 | Pus |
| 2245N0076 | 06Y117 | Taiwan | 2006 | 59 | 59 | Pus |
| 2245N0077 | 06Z044 | Taiwan | 2006 | 59 | 59 | Pus |
| 2245N0078 | 06Z135 | Taiwan | 2006 | 59 | 59 | Pus |
| 2245N0079 | 08S145 | Taiwan | 2008 | 59 | 59 | Blood |
| 2245N0080 | 10J010 | Taiwan | 2010 | 59 | 59 | Wound/Pus |
| 2245N0081 | 10J228 | Taiwan | 2010 | 59 | 59 | Ascites |
| 2245N0082 | 10K153 | Taiwan | 2010 | 59 | 59 | Sputum |
| 2245N0083 | 10M060 | Taiwan | 2010 | 59 | 59 | Sputum |
| 2245N0084 | 10T224 | Taiwan | 2010 | 59 | 59 | Blood |
| 2245N0085 | 10V026 | Taiwan | 2010 | 59 | 59 | Pus/Skin |
| 2245N0086 | 10Y144 | Taiwan | 2010 | 59 | 59 | Wound |
| 2245N0087 | 10Z202 | Taiwan | 2010 | 59 | 59 | Wound/Pus |
| 2245N0088 | 10Z213 | Taiwan | 2010 | 59 | 59 | Blood |
| 0864N0011 | 00.3468.G | UK | 2000 | 59 | Putative ST59 | Neck |
| 0864N0012 | 00.10399.P | UK | 2000 | 59 | Putative ST59 | Right Leg |
| 0864N0013 | 05.3403.N | UK | 2005 | 59 | Putative ST59 | Urine |
| 0864N0028 | H062820564 | UK | 2006 | 59 | 59 | Extremely bad wound infection |
| 0864N0030 | H064620454 | UK | 2006 | 59 | 59 | Skin infection |
| 0864N0014 | 06.1907.K | UK | 2006 | 338 | Putative ST59 | Breast |
| 0864N0029 | H063520132 | UK | 2006 | 338 | 59 | 0 |
| 0864N0031 | H071560489 | UK | 2007 | 59 | 59 | Skin abscess following burn |
| 0864N0033 | H074840490 | UK | 2007 | 59 | 59 | Infected wound |
| 0864N0039 | 07.7540.V | UK | 2007 | 59 | Putative ST59 | Wound Swab |
| 0864N0032 | H073700351 | UK | 2007 | 338 | 59 | Cutaneous abscess |
| 0864N0042 | 08.7522.S | UK | 2008 | 59 | Putative ST59 | Aspirate |
| 0864N0041 | 08.5366.V | UK | 2008 | 375 | Putative ST59 | Blood |
| 0864N0034 | H092100245 | UK | 2009 | 59 | 59 | C-section wound |
| 0864N0035 | H094300322 | UK | 2009 | 59 | 59 | Toe wound |
| 0864N0043 | 09.3149.P | UK | 2009 | 59 | Putative ST59 | Pustule(s) |
| 0864N0044 | 09.7352.Bb | UK | 2009 | 59 | Putative ST59 | Blood |
| 0864N0036 | H101480426 | UK | 2010 | 59 | 59 | Bacteraemia |
| 0864N0040 | H102040298 | UK | 2010 | 59 | 59 | Bacteraemia, soft tissue cellulitis |
| 0864N0045 | 10.5641.M | UK | 2010 | 59 | Putative ST59 | Nose, Axilla |
| 0864N0046 | 10.7639.K | UK | 2010 | 59 | Putative ST59 | Blood |
| 0864N0095 | H101980243 | UK | 2010 | 59 | 59 | Wound |
| 0864N0096 | 11.5376.D | UK | 2011 | 59 | Putative ST59 | Blood |
| 0864N0015 | 11.5388.K | UK | 2011 | 375 | Putative ST59 | Blood |
| 0864N0006 | 1500 | USA (Midwest) | 2004 | 59 | 59 | Folliculitis |
| 0864N0007 | 1699 | USA (Midwest) | 2004 | 59 | 59 | Empyema |
| 0864N0009 | 2032 | USA (Midwest) | 2004 | 59* | 59 | Sinusitis |
| 0864N0008 | 2551 | USA (Midwest) | 2005 | 59 | 59 | Line Infection |
| 0864N0010 | 2851 | USA (Midwest) | 2005 | 59 | 59 | Skin Colonisation |
| 0864N0088 | 4078 | USA (South) | 2005 | 59 | 59 | Bacteraemia |
| 0864N0090 | 2416 | USA (Midwest) | 2005 | 59 | 59 | Post-op Wound Infection |
| 0864N0091 | 2653 | USA (Midwest) | 2005 | 59 | 59 | Abscess |
| 612_32 | NARSA676 RT USA | USA (Northeast) | 2005 | 59? | 59 | 0 |
| 0864N0005 | 13139 | USA (Midwest) | 2008 | 59 | 59 | Skin Colonisation |
| 0864N0001 | 9101327 S-1 | USA (Midwest) | 2009 | 59 | 59 | Nasal Colonisation |
| 0864N0003 | 9113004 S-1 | USA (Midwest) | 2009 | 59 | 59 | Hair brush |
| 0864N0002 | 9171322 S-1 | USA (Midwest) | 2010 | 59 | 59 | Throat Colonisation |
| 0864N0004 | 8116028 S-1 | USA (West) | 2010 | 59 | 59 | Throat Colonisation |
| 0864N0087 | 9138302 S-1 | USA (Midwest) | 2010 | 59 | 59 | Bathroom door handle |
| 2245N0055 | 9079634 S-1 | USA (Midwest) | 2010 | 59 | 59 | Throat Colonisation |

**Table S1: 120 ST59 isolates included in phylogenetic analysis.** Strains were obtained from different countries around the world, as indicated by ‘country’. Isolates were given their own identifiers in our study (‘Sample ID’); the original names of the strains are also presented here (‘Lab ID’) and the year in which the isolate was sampled is reported. The results of multi-locus sequence typing (‘ST’) in both the lab and on the computer (‘*in silico*’) are reported, along with information about the source of the sample, where available.
